# Systematic *in vitro* comparison of decellularization protocols for blood vessels

**DOI:** 10.1371/journal.pone.0209269

**Published:** 2018-12-17

**Authors:** Robin Simsa, Arvind Manikantan Padma, Philipp Heher, Mats Hellström, Andreas Teuschl, Lachmi Jenndahl, Niklas Bergh, Per Fogelstrand

**Affiliations:** 1 VERIGRAFT AB, Gothenburg, Sweden; 2 Department of Molecular and Clinical Medicine/Wallenberg Laboratory, University of Gothenburg and Sahlgrenska University Hospital, Gothenburg, Sweden; 3 Laboratory for Transplantation and Regenerative Medicine, Department of Obstetrics and Gynecology, Sahlgrenska Academy, University of Gothenburg, Gothenburg, Sweden; 4 Ludwig Boltzmann Institute for Experimental and Clinical Traumatology/AUVA Research Center, Vienna, Austria; 5 Austrian Cluster for Tissue Regeneration, Vienna, Austria; 6 Department of Biochemical Engineering, UAS Technikum Wien, Vienna, Austria; Politecnico di Milano, ITALY

## Abstract

Decellularization of native blood vessels is a promising technology to generate 3D biological scaffolds for vascular grafting. Blood vessel decellularization has been performed in previous studies under various experimental conditions, that complicates comparison and optimization of suitable protocols. The goal of this work was to systematically compare the decellularization and recellularization efficacy of 5 different protocols utilizing the detergents sodium dodecyl sulfate (SDS), sodium deoxycholate (SDC), CHAPS and TritonX-100 together with DNA-removing enzymes on porcine vena cava in a perfusion bioreactor setup. Additionally, we tested the effect of DNase on the extracellular matrix (ECM) properties. We found that all protocols could efficiently decellularize blood vessels. Mechanical strength, collagen preservation and ECM integrity were similar among all tested detergents, yet TritonX protocols required long-term DNase application for complete decellularization. However, TritonX-based protocols showed the greatest recellularization efficacy with HUVECs *in vitro*. Furthermore, we developed a novel protocol for TritonX which improved recellularization and reduced total process time and ECM stiffness compared to previous protocols. SDS, SDC and CHAPS based protocols had a lower recellularization potential. In conclusion, decellularization of blood vessels can be achieved with all tested reagents, but TritonX treated ECM can be most efficiently recellularized with endothelial cells.

## Introduction

Vascular diseases such as atherosclerosis or chronic venous insufficiency are wide-spread around the world and have a great impact on international health expenses [1,2]. A common treatment option is the replacement of the diseased vessel with a vascular graft. Typically, two types of vascular grafts have been widely employed in vascular surgery–autologous grafts and synthetic grafts. Autologous grafts are the current gold standard of vascular grafting, utilizing the patients’ own veins and arteries as graft conduits. However, not all patients have suitable vessels for autologous grafting and the harvest of vessels adds time to the surgical procedure and may cause donor site morbidity, especially in elderly patients [3]. The second choice is synthetic grafts such as polytetrafluoroethylene (PTFE) or polyethylene terephthalate (Dacron). Synthetic grafts are readily available “off-the-shelf” and reliable as large diameter vessel replacement (>6mm). However, poor patency results due to thrombosis are an issue in small diameter vessels (<6mm). Limited growth potential and non-biodegradability is an additional problem, especially for pediatric patients that require dynamic grafts [4].

There have been numerous attempts to find suitable alternative grafts using tissue engineering. Tissue engineered vascular grafts (TEVGs) are based on biological materials and scaffolds [5,6]. A promising approach to acquire TEVGs is decellularization (DC) of native blood vessels, which has become a major research field in vascular engineering [7–14]. DC grafts utilize the extracellular matrix (ECM), which serves as a scaffold with similar structure and mechanical properties as the native vessel. To reduce the risk of adverse immune responses, all cells in the donor’s vessel must be completely removed. Ideally, a vascular graft based on DC should withstand physiological blood pressure, have low immunogenicity, and be non-thrombogenic. To reduce the risk of thrombosis, recellularization with endothelial cells—either *ex vivo* before implantation or *in situ* after implantation—is regarded necessary for a long-term functional vascular graft. The endothelium prevents blood clotting and protects against the formation of intimal hyperplasia and graft atherosclerosis [15,16].

The most commonly used DC strategies include physical, chemical and/or enzymatical methods. These need to be carefully adjusted to keep a proper balance between removal of cellular material and preservation of ECM integrity. DC protocols can furthermore influence the immunogenicity of the ECM, which can be a risk factor for clinical applications [17]. DC reagents include ionic, non-ionic and zwitterionic detergents, which disrupt interactions between cells, lipids, proteins and DNA [15]. Ionic detergents such as SDS and SDC are effective in cell removal, yet might disrupt the ECM structure and fully denature proteins [18]. Non-ionic detergents such as TritonX-100 (TX) are less harsh on the ECM and maintain native protein structure. However, TX disrupts interactions between lipids, proteins and DNA, and the reported DC efficacy of TX varies [19]. Zwitterionic detergents such as 3-[(3-cholamidopropyl) dimethylammonio]-1-propane sulfonate (CHAPS) and tri-n-butyl-phosphate (TNBP) have properties of both ionic and non-ionic detergents and show good ECM ultrastructure preservation, but limitations in complete cell removal [15,18]. Following detergent application, enzymatic treatment with endonucleases (e.g. DNase) or exonucleases (e.g. Benzonase) is common to cleave nucleotide bonds and remove remnant DNA. Multiple protocols for DC of blood vessels have been published, however the results are conflicting due to varying experimental conditions, which makes direct comparison of different protocols difficult [20]. Hence, there is a need for a systematic, standardized comparison of advantages and disadvantages of DC protocols to be able to choose an ideal protocol for DC.

The aim of this study was to compare different DC protocols of blood vessels with a focus on cell removal, mechanical strength, ECM integrity, and cell seeding capacity. The protocols are based on 4 established methods for decellularization [10,21–24] and one novel protocol. The protocols utilize the most commonly used detergents for DC. They were all tested on porcine vena cava in a perfusion bioreactor setup.

## Materials and methods

### Chemicals and reagents

*Abcam* (Cambridge, UK): Calcein AM; *Bionordika* (Stockholm, Sweden): EGM-2 SingleQuot Kit Suppl. & Growth Factors; *MerckMillipore* (Massachusetts, USA): Triton X-100, TNBP, EDTA; *Promega* (Wisconsin, USA): Tris Base, CellTiter 96 AQueous One Solution Cell Proliferation Assay (MTS); *Protean* (Dobra Voda, Czech Republic): Benzoase; *Roche* (Switzerland): MTT Reagent; *Scharlab* (Barcelona, Spain): Sodium acetate; *Sigma* (Missouri, USA): Sodium Deoxycholate, Acetylsalicylic acid, PBS -/-, PBS +/+, MgCl2, Tween 20, Sodium citrate tribasic dihydrate, Collagenase from Clostridium histolyticum, Endothelial Growth Medium, Papain from papaya latex; *Thermo Fisher Scientific* (Massachusetts, USA): Sodium Dodecyl Sulfate, Antibiotic-Antimycotic (Penicillin, Streptomycin, Amphotericin), CHAPS, NaCl, L-cysteine HCl, DAPI; *VWR* (Pennsylvania, USA): DNase; *3Helix* (Utah, USA): Collagen Hybridizing Peptide (F-CHP).

### Animals and harvesting of veins

Porcine vena cava were obtained from the Slaughterhouse KLS Ugglarps AB in Dalsjöfors, Sweden. The vessels were harvested approximately 30 minutes after killing of the pigs. Vena cava were dissected, cleaned from excess tissue, and stored in PBS containing 0.5% antibiotic-antimycotic (AA) for approximately two hours. The average length of the veins was 58.33 mm ± 7.76 and average diameter was 16.11 mm ± 1.54. The veins were then frozen in sterile PBS + 0.5% AA at -80 °C for decellularization. Freshly harvested veins were used as native controls for histological analysis and DNA quantification. Freeze-thawed veins were used as native controls for all other experiments.

### Decellularization methods

**Fig 1:**
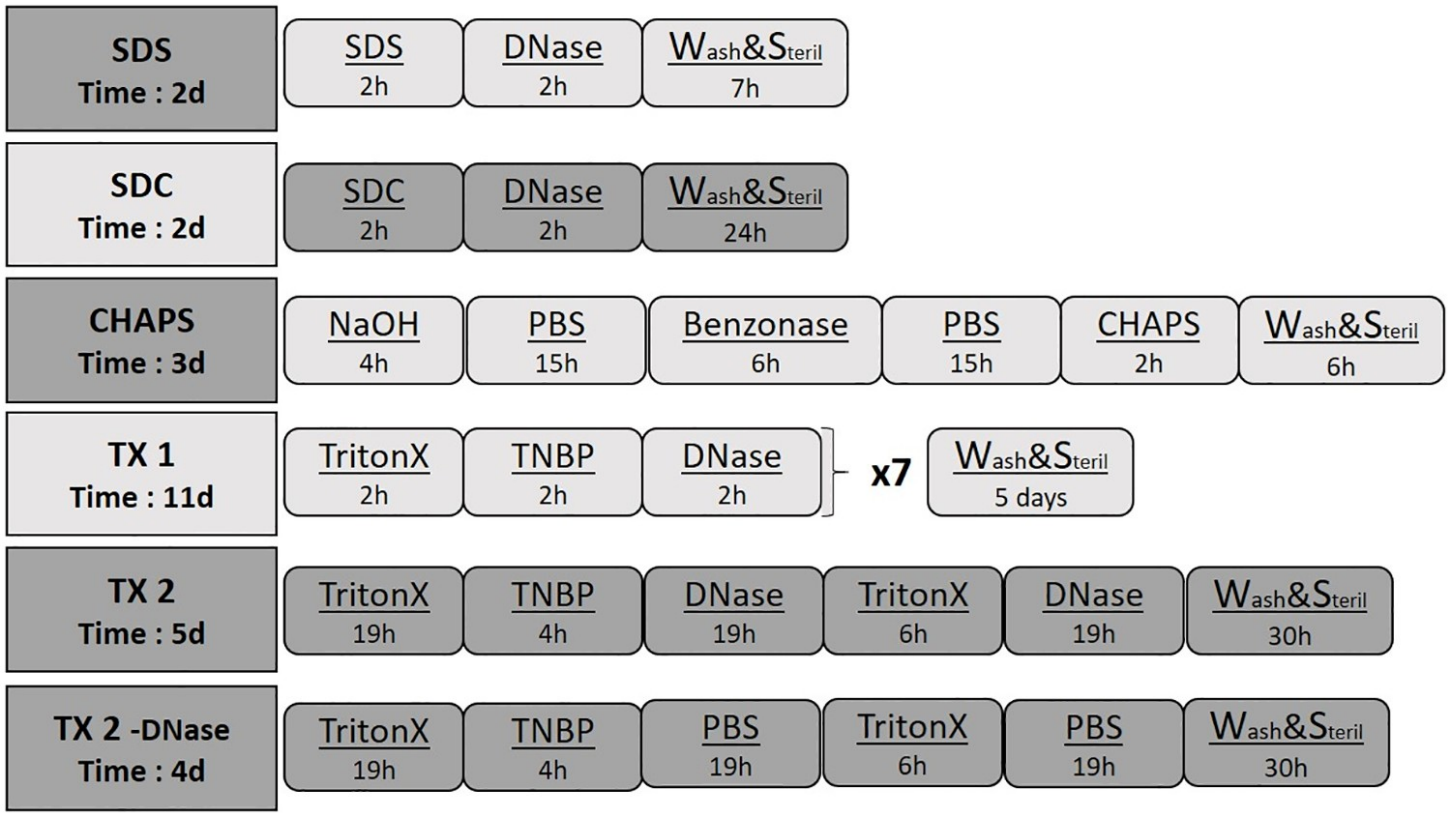
Schematic illustration of decellularization protocols. Concentration of reagents was SDS 0.1%, SDC 1%, NaOH 0.1M, CHAPS 8mM, TritonX 1%, TNBP 1%, DNase 40 U/mL and Benzonase 2 U/mL. Underneath the abbreviation for each protocol, the total process time for a single vein decellularization is noted. The group TX 2 is a novel, shorter TX-based protocol, and the group TX 2 -DNase was treated as the TX 2 group, but without enzyme application.

Previously published protocols utilizing 1% TX and 1% TNBP[24], 0.1M NaOH and 8 mM CHAPS[22], 0.1% SDS[10], 1% SDC [21] as well as one new protocol utilizing TX and TNBP were chosen for the DC experiments, with or without combination with the endonuclease DNase or the exonuclease Benzonase (Fig 1). Decellularization was performed at a steady temperature (37°C), agitation (115 rpm) and perfusion (100 mL/min) following an initial freeze-thaw step (see above). Between different detergent applications, veins were washed 5–10 min in dH2O or PBS. Detergent and washing buffer volume was 200 mL, enzyme application volume was 40 mL for all groups. Following primary decellularization, veins were washed multiple times in PBS and sterilized for 1h in 0.1% peracetic acid, followed by a final wash. Then luers were disconnected from the vein, tissue samples taken for further analysis and the rest of the vein frozen in PBS containing 0.5% AA. Incubation times and detergent concentrations were applied as in the original protocols (Fig 1). Summary of the methods is given below. SDS: Incubation in 0.1% SDS for 16h. Washing and incubation continued in DNase (40 U/mL) for 2 h, followed by final wash and sterilization(7h). SDC: Incubation of vein in 1% SDC for 2h. Washing and incubation continued in DNase (40 U/mL) for 2 h, followed by final wash and sterilization (24h). CHAPS: Incubation of vein in a solution containing 0.1M NaOH, 1M NaCl and 25 mM EDTA for 4h. Veins were washed and incubated in a buffer containing 100 mM EDTA o/n, then washed and incubated in a solution containing 2 U/mL Benzonase, 47 mM Tris, 1.4 mM MgCl2 and 19 mM NaCl for 6h, followed by washing and another incubation in EDTA buffer o/n. Washing and incubation of vein continued in a solution containing 8mM CHAPS, 1 M NaCl and 25 mM EDTA for 2h, followed by final wash and sterilization (6h). TX 1: Incubation of vein in Triton X (1%) for 2h, followed by washing and incubation in TNBP (1%) for 2h, and washing and incubation in DNase (40 U/mL) for 2h. Veins were then stored in EDTA solution o/n. This procedure was repeated for 7 cycles, followed by final was and sterilization (5 days). TX 2: Incubation of vein in Triton X (1%) for 19h, followed by washing and incubation in TNBP (1%) for 4h. Washing and incubation in DNase (40 U/mL) for 19h. Washing and incubation in TritonX (1%) for 6h. Washing and incubation in DNase (40 U/mL) for 19h, followed final wash and sterilization (30h). TX 2 -DNase: Same protocol as for TX 2 was applied, but instead of DNase, veins were incubated in PBS.

### Perfusion bioreactor setup

**Fig 2:**
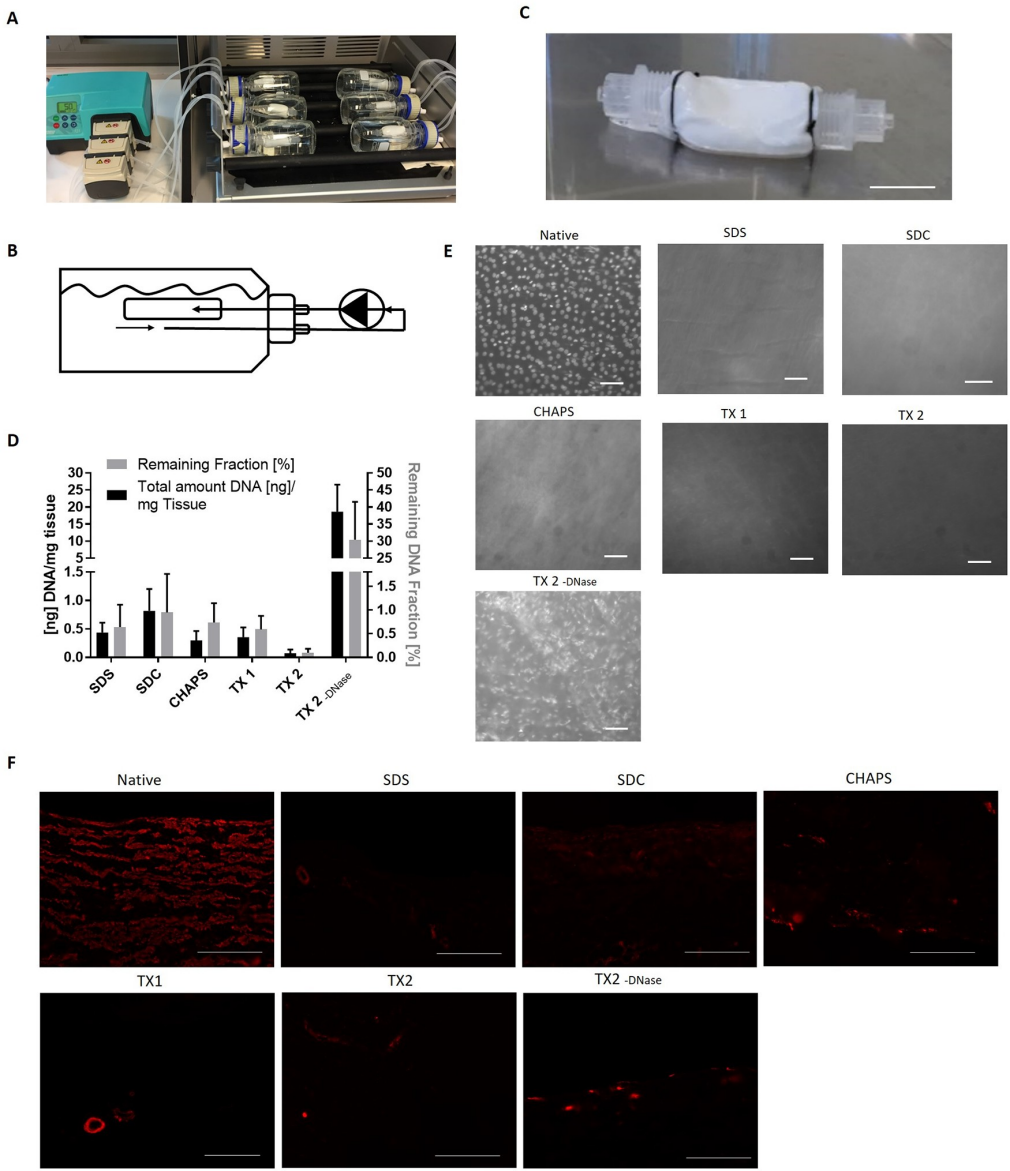
Decellularization efficacy. Porcine vena cava were decellularized according to the respective protocols and DNA content was measured. (A) Setup of the bioreactor, with vena cava inside 250mL flasks connected with tubing system to peristaltic pumps. (B) Schematic overview of perfusion through vena cava, allowing circulating liquid flow. (C) Illustrative picture of a decellularized vein connected to luers. Scale bar represents 2.5 cm. (D) DNA in the ECM was quantified and the result shown as ng DNA/mg tissue (left y-axis) and as % of remaining DNA compared to the DNA content of the same native blood vessel prior to decellularization (right y-axis) (100%, n = 4). Both Y-axes were divided into 2 sections to show small differences in the DC groups. (E) DAPI stained double-stranded DNA on the luminal side of ECM of the native blood vessel and the DC groups. Scale bar represents 50 μm. (F) Staining of paraffin-embedded sections with anti-alpha smooth muscle actin. Scale bar represents 100 μm.

A perfusion bioreactor system was designed, utilizing a peristaltic pump (030.3134.3DE, Watson Marlow, Wilmington), an incubation shaker (KS 4000i control, IKA, USA) and 250 mL flasks (Duran Flasks, Sigma) (Fig 2A). The pumping system and the incubation shaker allowed a steady temperature, agitation and perfusion, while the flasks functioned as the main incubation unit for the blood vessels. Lids with 2 insert holes were used to allow connection of the samples to the pumping system with commercially available tubes (Nalgene silicon tubing, VWR) (Fig 2B). Prior to decellularization, all veins were tied to female luer connectors (Cole Parmer, USA) with 0–2 mm sutures (Ethicon, Sweden) and attached to the tubing system (Fig 2C). The output of the tubing system led through the vein, while the input remained at the bottom of the flask, creating a circulating liquid flow. When less volume usage was desired, a 60 mL flat bottom tube was put inside the flask.

### Sterility test

Following decellularization, 200 μL of the veins′ storage buffer was added to 2 mL Luria Broth for incubation at 37°C for 5 days. OD was measured at 600 nm with Evolution 60s UV-visible Spectrophotometer (Thermofisher, USA). Potential bacterial contamination was determined by comparison against a negative control (Luria Broth without added components).

### DNA quantification

DNA was extracted using the DNeasy Blood and Tissue kit (Qiagen, Germany) and quantified with the Qubit dsDNA HS assay kit (Life technologies, USA). Briefly, 10–25 mg tissue both from the starting material prior to decellularization and from the decellularized samples were treated with proteinase K to quantify the total amount of DNA/mg wet tissue and to calculate the percentage of DNA removal after DC. Measurement of absorbance was performed with the Qubit 3.0 Fluorometer (ThermoFisher, USA). The detection limit of DNA was 0.2 ng/μg.

### Histology staining

DAPI (4',6-diamidino-2-phenylindole) staining was performed *en face* by incubation of tissue sample (luminal side facing upwards) with 25 μg/mL DAPI diluted in PBS for 2 min in the dark. When tissue was very thick, adventitia and media layer were removed similar to the approach described by Jelev et al. [25] ECM was fixed on a plate with forceps under a microscope. Intima layer was slowly removed by stretching with forceps and simultaneous cutting on the edges with a scalpel, followed by recovery in PBS.

DAPI, Hematoxylin-Eosin (H&E), Masson Trichrome (MTC), Alcian Blue (AB) and Verhoeff van Gieson (VvG) and Periodic Acid-Schiff (PAS) stains were also applied on paraffin-embedded cross sections (5 μm thickness), following standard protocols for fixation, dehydration, embedding, cutting, deparaffination, rehydration and staining.

### Immunohistochemistry

Immunohistochemistry staining was performed on paraffin-embedded samples, following rehydration and antigen retrieval in a water bath at 95°C in a Tris-EDTA buffer at pH 9. Samples were blocked with 10% FBS and stained with anti-Laminin (1:100, ab11575, Abcam, UK) or anti-alpha smooth muscle actin (1:75, ab7817, Abcam, UK) at 4°C in wet chamber o/n, following by incubation with respective secondary antibody at RT for 1h.

### ECM components

The ECM components collagen (soluble and insoluble) and glycosaminoglycans (GAGs) were quantified with commercially available assay kits (Biocolor, UK), following the supplied protocols, using 15–30 mg wet tissue weight as starting material for the respective extraction.

### Biomechanical tests

Mechanical ringlet tests were performed similarly to a method published by Schneider and colleagues [26] to obtain Young′s modulus (elastic modulus; measures elasticity of a material), maximum tensile strength (F_max_), burst pressure and tissue deformation at maximum tensile strength. A custom-made device was constructed for cutting the veins into equally sized rings (S3 Fig). Prior to testing, wall thickness (with a digital caliper) and length of the vein rings was measured. Outside diameter of the specimen was calculated from its half circumference. The vein rings (width 6 mm) were then separately loaded between 2 steel rods and clamped vertically into the testing machine (ZwickiLine, Zwick/Roell, Germany). Force was applied until failure with a rate of 10 mm/min. Young′s modulus was calculated with the supplier’s software from linear phase of stress-strain curve (S4 Fig) starting from 2% dilation. Burst pressure was obtained from applying Barlow′s formula:

$$p=\frac{2*F_{max}*t}{D}$$

With p being the burst pressure, F_max_ the force at failure, t the vessel thickness and D the outside diameter of the material.

### Biodegradability assay

Biodegradation was performed as described previously [27,28]. Briefly, 10–15 mg of lyophilized tissue samples were incubated at 37°C with gentle shaking of 70 rpm in 500 μL of 0.1% Collagenase from *Clostridium histolyticum* (Sigma, USA) diluted in PBS. Control samples were incubated only in PBS. At 4 different time points up to 48h, samples were removed, lyophilized once more and weighed. Biodegradation, given as remaining tissue fraction, was calculated with the following equation:

$$Remaining\;fraction\;(\%)=100*\frac{W_{0}}{W_{t}}$$

where W_0_ represents the initial weight and W_t_ the measured weight of the tissue sample at time point t.

### Collagen hybridizing peptide analysis

Fluorescent Collagen Hybridizing Peptide (F-CHP) was applied, following the instructions of the supplier (3Helix, USA)[29]. Briefly, paraffinized tissue sections (5 μm) were rehydrated and stained with F-CHP. 20 μM F-CHP in PBS was first heat-activated at 80°C for 5 min, subsequently cooled in an ice-water bath for 60 sec and immediately applied on the tissue sections. As a negative control, PBS and non-heat activated F-CHP was used. A positive tissue control was generated by boiling tissue sections in a pressure heater for 3 min prior to staining. After incubation o/n at 4°C, a cover-glass was mounted and samples were visualized in the green channel of a fluorescent microscope. Pictures were taken with constant microscope settings for emission time and magnification, allowing direct comparison between samples. (Axiovert 40 CFL, Carl Zeiss, 10x objective).

Fluorescence intensity was compared between the samples using the software ImageJ (v1.51, NIH), similar to the method described by McCloy et al [30]. Firstly, up to 5 regions of interest on the ECM per picture were selected (all with the same area size) and area, mean intensity and integrated density was measured. This was repeated for multiple pictures for one sample. Also, at least 2 areas of the background outside of the ECM area were measured (area size not relevant). Fluorescence intensity was calculated with following formula:

FluorescenceIntensity=Averageintegrateddensityofsample–(areaofsample*averagemeanintensityofbackground)

Average fluorescence intensity of the unspecifically stained sections was subtracted from the fluorescence intensity of the specifically stained sample. Data were normalized to the fluorescence intensity of the native sample.

### Cell cultures

Human umbilical vein endothelial cells (HUVECs) from ATCC (cat. No. CRL-1730, Manassas, USA) were used for the cell seeding experiment (passage 5) in MCDB 131 Medium (Thermo Fisher, USA) supplemented with endothelial growth medium kit (CC-4176, Bionordika, Sweden) and Antibiotic-Antimycotic (15240062, Thermo Fisher, USA). For the cytotoxicity experiment, human embryonic kidney (HEK) 293 cells were used in DMEM (10569010, Thermo Fisher, USA) supplemented with 10% FBS (10500064, Thermo Fisher, USA) and Antibiotic-Antimycotic (15240062, Thermo Fisher, USA). Incubation was performed at 37°C, 5% CO2.

### Cytotoxicity assay

Toxic effects of the ECM and potential residual detergents was determined with an MTT assay on HEK cells, which is an established cell line system for cytotoxicity assays [31,32]. 1*10^4^ HEK 293 cells were seeded with 100 μL media onto a 96 well plate o/n, then 20 mg wet tissue pieces were added to cells in triplicates for each vessel and incubated for 36 h. 10 μL MTT reagent was added to media and incubation continued for 6 h. Then, 100 μL SDS was added and incubated o/n. Absorption was read at 550–600 nm. The control consisted of the same cell number seeded without tissue.

### Recellularization assay

The viability of HUVECs seeded onto the decellularized ECM was determined by MTS assay as well as Calcein AM staining, as described previously [26,33,34]. Decellularized vessels were cut into 5 mm x 5 mm sections and placed in a 24 well plate with the luminal side facing upwards. Endothelial growth media was added to equilibrate the sections for 30 min at 37°C, followed by removal of the media and careful drying of the surfaces with a sterile swab. 75.000 HUVECs in 15 μL media were then seeded onto each section and were allowed to attach for 1h at 37°C with 400 μL media at the bottom of the wells to keep the tissue moist. 1.6 mL media was added afterward, and samples incubated at 37°C for 5 days, with media change every 2 days.

MTS assay was performed by aspirating media from the 24 well plates and subsequent incubation with media containing MTS solution in a ratio of 6 to 1 for 1.5 h at 37°C. Viable cells reduce MTS solution to a colored formazan product that is measured spectrophotometrically at 490 nm. A standard was prepared by adding MTS solution to HUVECs of known density seeded onto wells without ECM. Unseeded ECM was used as a negative control. Following MTS assay, sections were washed with PBS for 5 min and kept in media o/n to regenerate. To further visualize cell viability, media containing 3 μM Calcein AM was added for 30 min, 37°C, followed by counterstaining with DAPI. Pictures were taken in green and blue channel of Leica DM5500 fluorescent microscope.

### Statistical analysis

For all graphs and statistical calculations, GraphPad Prism 7 (GraphPad Software Inc., San Diego, USA) was used. Statistical significance was obtained by one-way ANOVA testing. p-values equal to or less than 0.05 was considered significant. The asterisk signs above graphs indicates the following p-values: (*) = p ≤ 0.05, (**) = p ≤ 0.01, (***) = p ≤ 0.001. Data are presented as mean ± standard deviation (SD).

## Results

### Study design

Five different DC protocols for the commonly applied detergents SDS [10], SDC [21], NaOH + CHAPS [22,23] and TX + TNBP [24] were evaluated on porcine vena cava (Fig 1). The original TX + TNBP protocol (TX 1) is time-consuming and work-intensive, therefore a shorter protocol with more intensive DNase treatment (TX 2) was evaluated. Furthermore, the TX 2 protocol was used also without DNase treatment (TX 2 -DNase), to evaluate the importance of nuclease treatment for DC (Fig 1), after observing poor cell removal with TritonX based protocols with brief DNase treatment (S1 Text, S1 Fig). The veins were incubated in a perfusion bioreactor at 37°C at constant temperature and flow rate to achieve uniform removal of cellular material throughout the vessel (Fig 2A and 2B). All samples showed to be sterile following DC (data not shown).

### Decellularization with SDS, SDC, CHAPS and TritonX in combination with long-term DNase application efficiently removed cells from porcine vena cava

To assess cell removal efficacy, double-stranded DNA was quantified as an indicator of remaining cellular material. All DC groups had significantly lower DNA content (p≤0.001, n = 4) compared to native tissue (80.34 ± 35.42 ng/mg wet tissue). Only the group TX 2 -DNase, which was not enzymatically treated with DNase, had a high DNA content (18.62 ± 7.94 ng DNA/mg tissue), while the same protocol with DNase treatment (TX 2) had a very low DNA content (0.354 ± 0.17 ng DNA/mg tissue). The groups SDS, SDC, CHAPS and TX 1 & 2 all showed less than 1% remaining DNA fraction compared to the native group (Fig 2D).

*En face* staining with DAPI confirmed the results from the DNA quantification and showed remaining DNA only in the TX 2 -DNase group. The other groups had no or few remaining DNA fragments (Fig 2E). Compared to the native tissue, positive DAPI staining in TX 2 -DNase showed an altered nuclear morphology (smeared, unordered), suggesting that detergents were able to lyse cells, but not completely remove all DNA content. Cross-sections stained with DAPI and Hematoxylin-Eosin (H&E) confirmed this notion (S1 and S2 Figs). Furthermore, staining for α-smooth muscle actin (ASMA) revealed no or little remaining ASMA within the medial vessel wall layer of the decellularized groups, indicating an efficient removal of cellular proteins (Fig 2F).

### ECM composition and morphology

**Fig 3:**
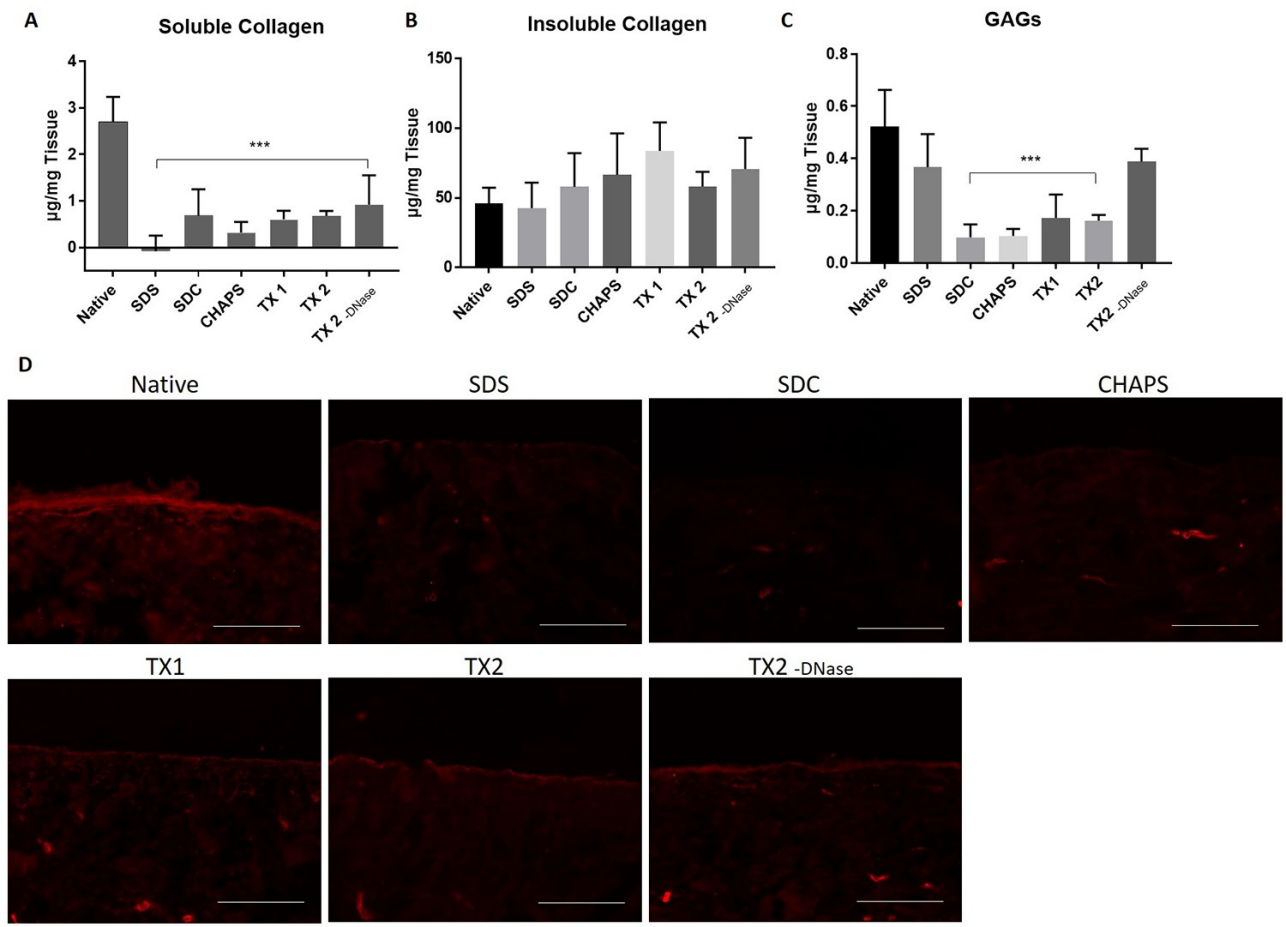
Quantification of the ECM proteins. Porcine vena cavas were decellularized according to the respective protocols and ECM proteins were analyzed using Biocolor assays and immunohistochemistry. (A) Quantification of soluble collagen. (B) Quantification of insoluble collagen. (C) Quantification of glycosaminoglycans (GAGs) (n = 3 in all groups). (D) Staining of paraffin-embedded sections with anti-laminin antibody. Scale bar represents 100 μm.

To observe the removal of common ECM proteins following DC, protein extraction and spectrophotometric measurement were performed for soluble and insoluble collagen and GAGs (n = 3). The amount of soluble collagen was significantly reduced in all groups compared to the native group (Fig 3A). No significant difference was observed between DC groups and native group in the amount of insoluble collagen (Fig 3B). GAG content was significantly decreased in all groups except in the groups SDS and TX 2 -DNase (Fig 3C). The basal membrane integrity was furthermore observed by laminin staining, which showed alterations in all DC groups compared to the native tissue (Fig 3D). The TX groups (TX 1 and TX 2) showed a slightly higher staining intensity compared to the SDS, SDC and CHAPS group. Histological staining to visualize collagen (MTC and H&E), GAGs (Alcian blue stain), polysaccharides and glycoproteins (PAS) and elastin (VvG stain) showed no obvious morphological alterations between groups (S2 Fig). Thus, only small overall differences between different DC groups in terms of ECM composition and morphology were detected, and TX groups showed higher retention of the basement membrane.

### Decellularized veins are biodegradable

**Fig 4:**
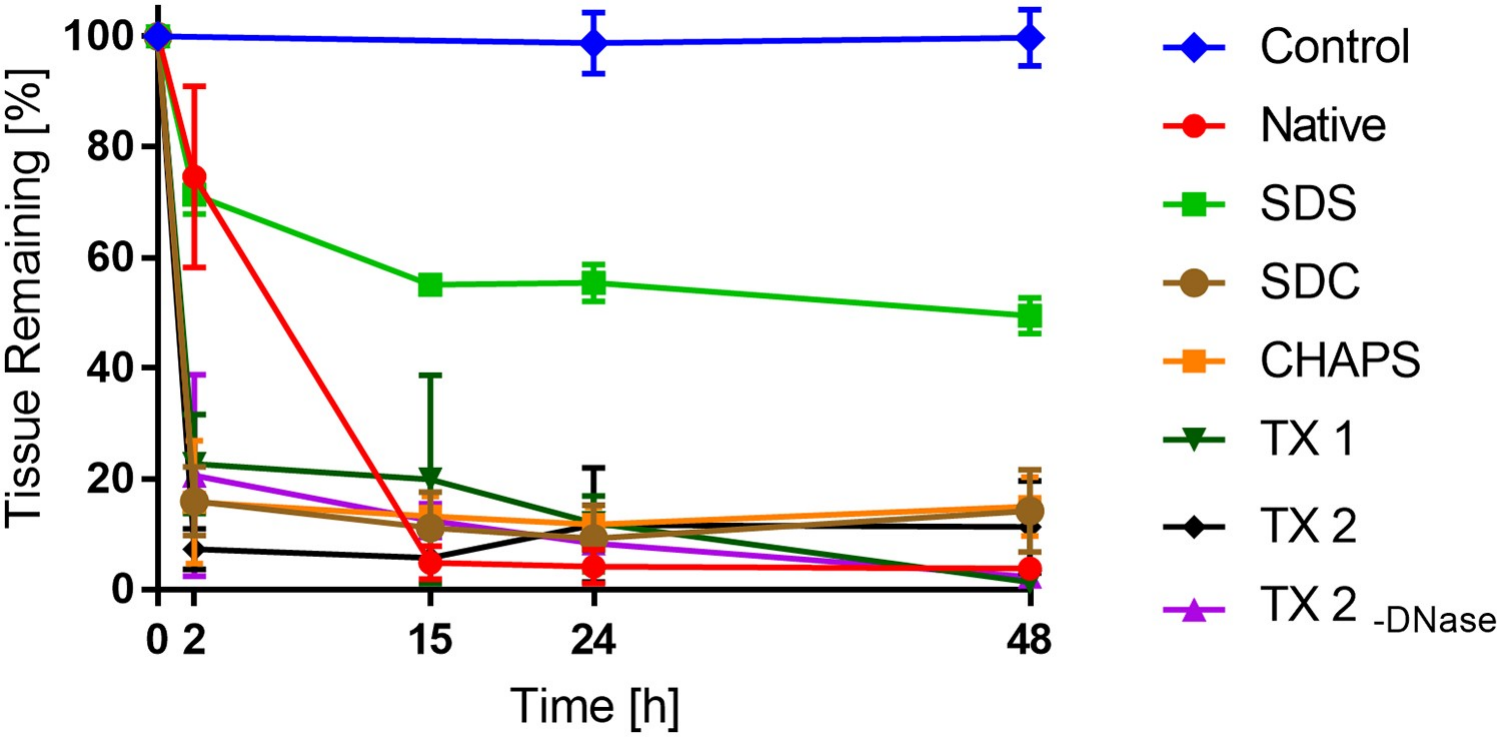
Assessment of tissue biodegradability. Biodegradability was measured by incubating lyophilized samples from decellularized or native porcine vena cava (n = 4 in each group) with 0.1% Collagenase I or PBS (negative control) and the tissue weight was measured at 0, 2, 15, 24 and 48h. The diagram shows the percentage of remaining tissue weight compared with time 0h.

An *in vitro* biodegradability experiment was performed to test the potential of the DC vessels to be remodeled by the patient’s own cells after implantation (Fig 4). Collagenase I treated DC (n = 3 per time point) and native tissue samples (n = 4 per time point) were weighed at different time points to determine the remaining fraction weight compared to the starting weight. After 2 h, a marked loss of tissue weight was observed in the SDC, CHAPS, TX 1 and TX 2 groups (<25% of remaining fraction, p<0.001 for all groups). In contrast, the SDS group was much less degraded (71 ± 7.3% remaining fraction, n.s.). The native group was also less degraded after 2h (75± 16% remaining fraction). At 15, 24 and 48h, the SDS group still had a high remaining fraction (49% ± 3.2 at 48h, p<0.001), while all the other groups, including the native group, showed low remaining fraction (<20%, p<0.001). These results suggest that all decellularized groups, except the SDS group, have a comparable biodegradability to the native group.

### Mechanical properties are similar to the native vessel

**Fig 5:**
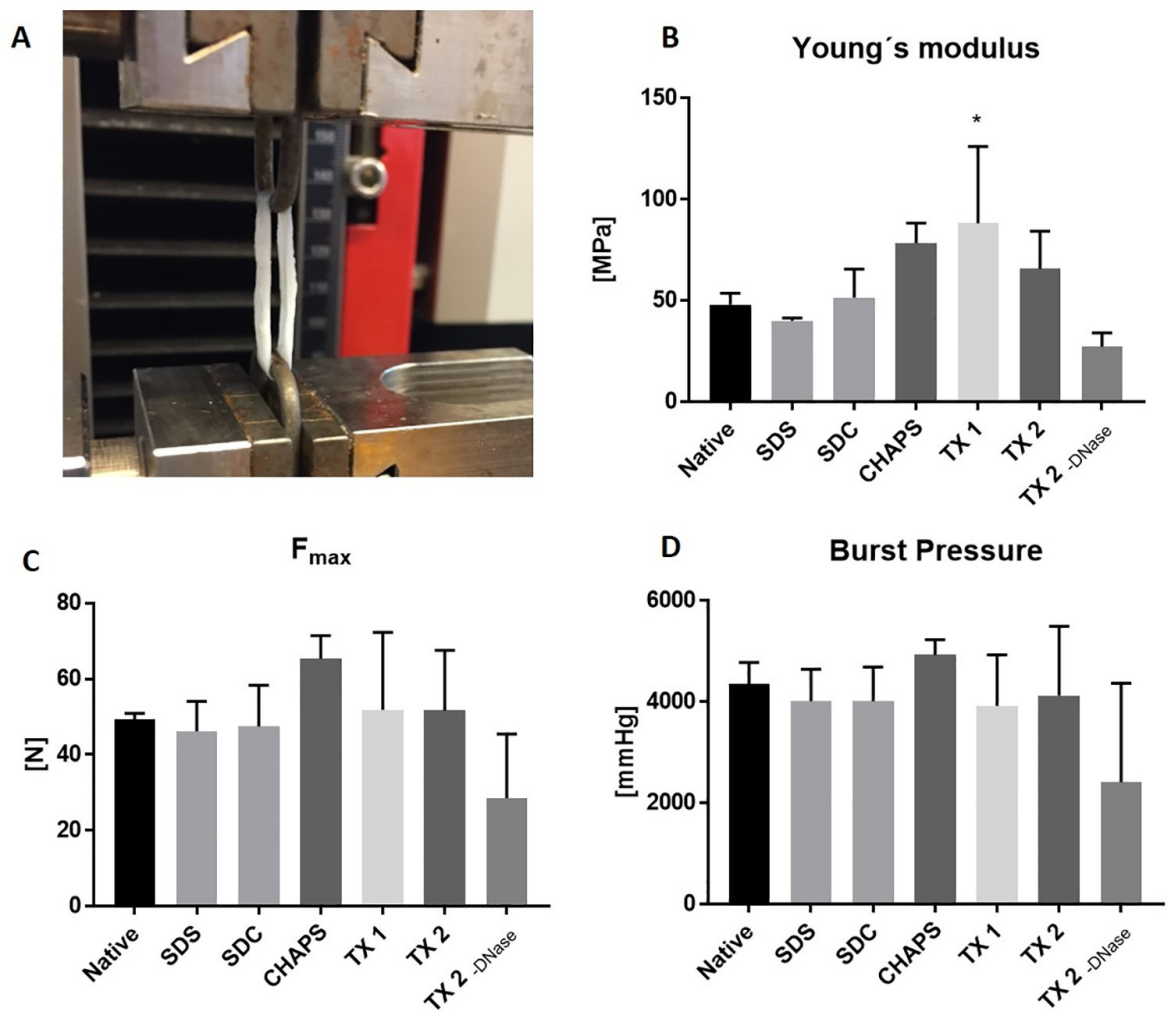
Mechanical testing of vein ringlets. Ringlets from decellularized and native vena cava were stretched until failure in a Zwick-Roell machine (A) Picture of testing setup with a vein ringlet clamped into the machine between 2 metal holders. (B) Measurement of material stiffness according to Young′s modulus. Higher values indicate increased stiffness. (C) Maximum tensile strength F_max_. (D) Theoretical burst pressure, calculated from Barlow’s equation (n = 3 veins, 3 ringlets from each vein as technical replicates).

To assess to effect of DC methods on mechanical properties, ringlets of decellularized vessels were strained until failure (Fig 5A) and analyzed for alterations in elasticity/stiffness (Youngs modulus), maximum tensile strength (F_max_), and burst pressure compared to native vessels (n = 3 vessels per group, 3 ringlets from each vessel as technical replicates).

The TX 1 group showed a significant increase in stiffness (88.15 ± 38.09 MPa, p = 0.035) compared to the native samples (48.07 ± 5.58 MPa, Fig 5B). The other groups did not show significant differences. There were no significant difference in maximum tensile strength (F_max_, Fig 5C) or burst pressure (Fig 5D) when comparing DC groups to the native group. Furthermore, a decreased deformation at F_max_ was observed in the groups TX 1 & 2 and CHAPS (S3 Fig). In conclusion, decellularized vessels had a similar tensile strength and burst pressure compared to the native vessel, and increased stiffness was observed in the groups TX 1.

### Collagen denaturation

**Fig 6:**
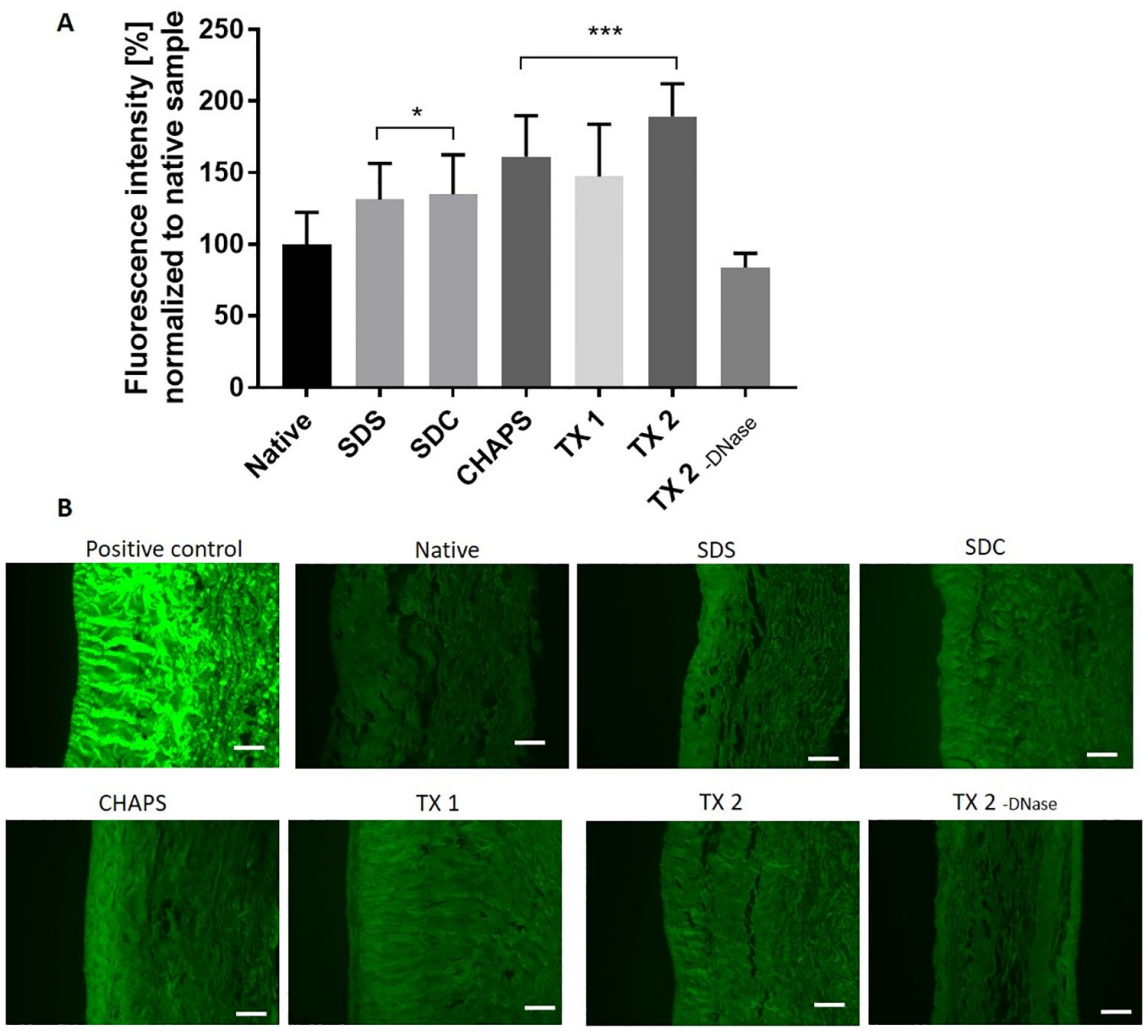
Detection of collagen denaturation. Tissue cross sections from DC and native porcine vena cava were incubated with a collagen hybridizing peptide labeled with fluorescein. Bound peptide was visualized under a fluorescence microscope. Multiple pictures per sample were taken in the green channel (488nm) under identical conditions (n = 4) and integrated density analyzed with ImageJ. (A) Fluorescence intensity is shown in average percentage of fluorescence compared to the native sample (100%). (B) Representative pictures of cross sections stained with the collagen hybridizing peptide. Pictures were taken with an exposure time of 5000 ms, except for positive control (boiled tissue section), which was visualized at 40 ms. Scale bar represents 100 μm.

To analyze collagen denaturation we incubated tissue sections with a collagen hybridizing peptide (n = 4) and quantified fluorescence intensity with ImageJ (Fig 6)[29,35].

The group TX 2 -DNase did not show significant changes of collagen degradation compared to the native sample. All other groups had increased signals from the collagen hybridizing peptide compared to the native sample, the highest in the groups TX 1, TX 2 and CHAPS. Even though differences between groups were found, changes in collagen degradation were minor overall. The positive control (boiled sample) was more than two magnitudes brighter than all other samples (the picture in Fig 6B were taken at an exposure time of 40 ms, while the other groups had an exposure time of 5000 ms).

### Biocompatibility of decellularized vessels with cells

**Fig 7:**
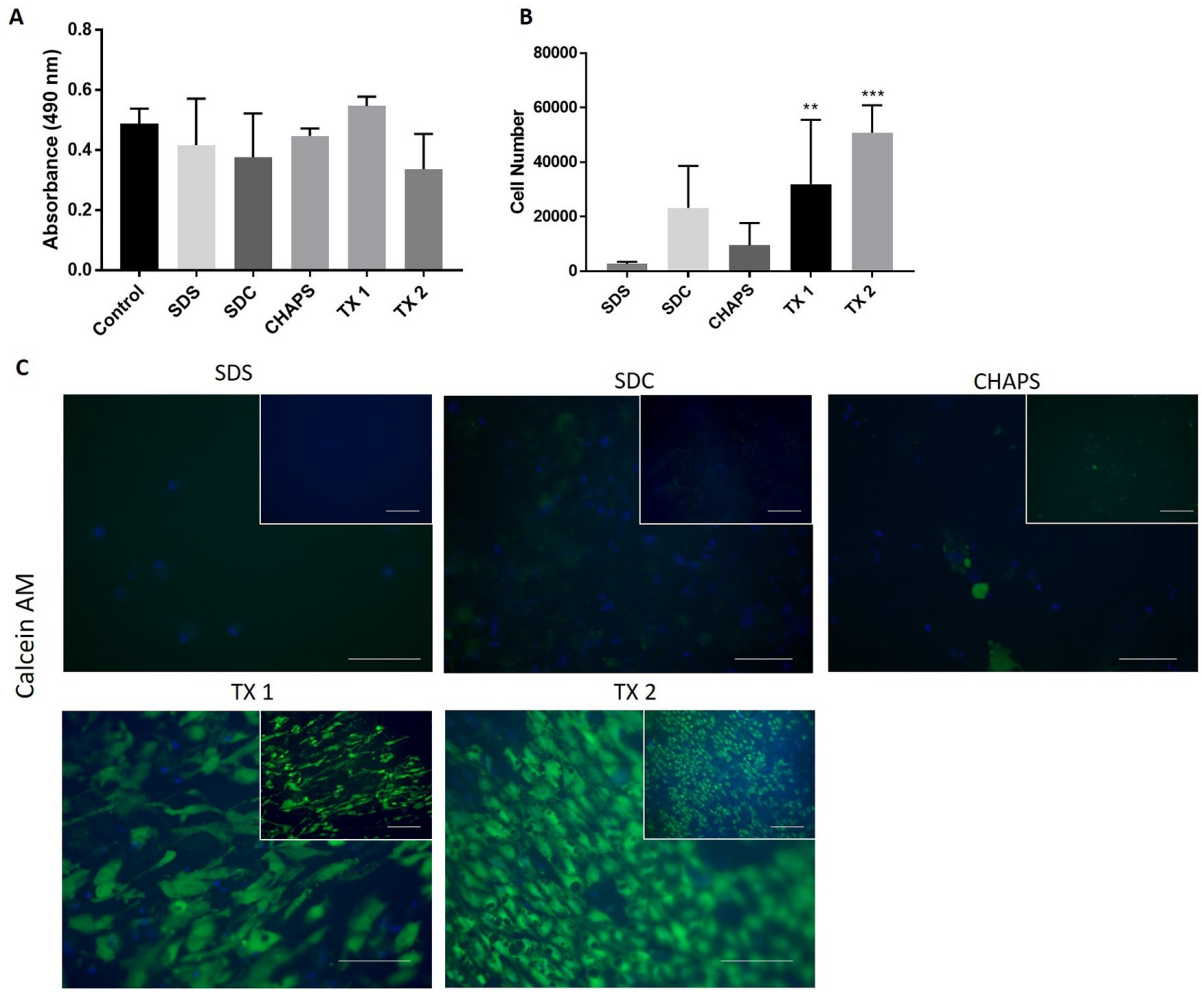
Cytotoxicity and *in vitro* EC seeding. Cell biocompatibility of decellularized samples was analyzed in a cytotoxicity and a cell seeding experiment. (A) Cytotoxic properties of samples from decellularized and native vessels were measured by adding tissue pieces to wells with pre-seeded HEK 293 cells. Cell viability was measured spectrometrically after 36h with an MTT assay.(B-C) Seeding with 75.000 HUVECs on the luminal side of the decellularized samples and incubation for 5 days. (B) Spectrometric analysis of the number of viable endothelial cells at day 5 with the MTS assay. (C) Visualization of alive endothelial cells with Calcein AM staining 5 days after. Pictures are taken in the green channel (488 nm) under identical microscopy conditions (n = 4 for all groups). Counterstaining was performed with DAPI. For observation of cell orientation and alignment as well as a general overview, pictures are presented as outlets (higher magnification) and inlets (lower magnification), scale bar equals 100 μm for both.

The groups with complete decellularization (SDS, SDC, CHAPS and TX 1 & 2) were tested *in vitro* for biocompatibility with cells. To assess the general cytotoxicity of decellularized vessels, tissue pieces of samples (n = 4) were added to cultured HEK 293 cells and cell survival was measured after 36 h using the MTT assay. We found no significant change in MTT absorbance signal in any group compared with controls (no tissue exposure). These results indicate that the decellularized tissues do not contain residual detergents or other harmful components at cell toxic levels (Fig 7A).

We next analyzed the ability of endothelial cells (HUVECs) to attach and grow onto the luminal surface of tissue pieces from decellularized groups (n = 4). Six days after seeding, cell viability was measured with the MTS assay. There was a significant increase in cell viability in the groups TX 1 & 2 compared to the groups SDS, SDC and CHAPS (Fig 7B), suggesting higher endothelial cell viability. This was further confirmed by Calcein AM staining (Fig 7C), where the groups TX 1 & 2 showed brightly stained cells on the luminal surface of the tissue pieces. Morphologically, the endothelial cells were elongated and aligned in the same direction. In the SDC and CHAPS groups only scattered cells with weak Calcein AM staining could be detected, indicating poor endothelial cell viability. The SDS-treated group showed no or few detectable endothelial cells. Thus, decellularization protocols with the mild detergents TX and TNBP (groups TX 1 & 2) were the most successful for cell seeding of endothelial cells *vitro*.

## Discussion

Decellularization of tissue is a method to generate 3D scaffolds for regenerative medicine applications. In this study, we systematically compared five different decellularization protocols for blood vessels regarding decellularization efficiency, ECM integrity and recellularization potential. Specifically, we have shown that all protocols efficiently removed cellular material. The recellularization potential was highest among the TX 1 and TX 2 groups. Furthermore, enzymatic removal of DNA showed to be very important for complete decellularization with TX. Differences in terms of biodegradation, biomechanical behavior, collagen denaturation and ECM protein retention were detected as well between DC and native groups. Taken together, decellularization can be obtained with a variety of reagents, but TritonX-based methods may be preferable for subsequent recellularization.

DC protocols for SDS [10], SDC [21], CHAPS [22] and TX [24] have previously reported successful decellularization of blood vessels. However, they were applied under varying experimental conditions regarding temperature, flow application (Static, agitation, perfusion), donor material (Human iliac, mammary or saphenous vein, rabbit vena cava, rat aorta) etc., which complicate comparisons. To allow comparison of the protocols and subsequent rational protocol design, we standardized the DC process by using steady parameters in a perfusion setup on porcine vena cava. The porcine vena cava is considered to be a relevant model to humans due to similar size, thickness and histological structure [9]. Indeed, we found that all DC reagents were suitable to completely decellularize the porcine vena cava in this standardized setup. To find a more time-efficient TX protocol, we additionally designed a novel TX protocol (TX 2) which reduced process time by more than 50% and was more resource-efficient compared to the original TX 1 protocol (S1 Table), yet was equally efficient in removal of cellular material and preservation of the ECM. The TX 2 protocol utilized DNase for long time periods. The importance of DNase for decellularization was investigated by directly comparing the protocol with and without DNase (TX 2/ TX 2 -DNase). We showed that TX 2 -DNase was not able to fully decellularize the blood vessel, but showed a higher mechanical stability and higher GAG content than the TX 2 group, suggesting an effect on these parameters by DNase application. These results are in contrast to previous publications, which do not mention negative effects of DNase on tissue [36,37]. The present study underlines that decellularization of blood vessels can be achieved with all tested protocols. Enzymatical treatment following TritonX application for complete removal of cellular material is essential, but negative effects on the ECM must be monitored.

Decellularized tissues have been used as a natural biomaterial for cell attachment, proliferation, differentiation and immune modulation for decades [38,39]. Preserving the complex protein structure and the stability of the ECM during decellularization is of vital importance for its function as a scaffold. In this study, we show that the different decellularization protocols preserved the general morphology, the collagen content and integrity of the ECM, but GAG content was reduced in most samples. GAGs such as keratan sulfate and heparan sulfate are present in the plasma membrane of cells [40] and therefore a reduction of GAGs after decellularization is expected. GAGs are also abundant in the ECM and have an important function in electrostatic binding of growth factors and cytokines. Hence the presence of GAGs is of importance for recellularization and can affect the grafts *in vivo* functionality [41,42]. Interestingly, the SDS group did not show reduced GAG content compared to the other groups. This might be attributed to the short time (2h) and concentration (0.1%) of SDS used. We also showed an alteration on the basement membrane, which has an important function for cell attachment and anti-immunogenicity. The basement membrane appeared less altered in the TX groups. This confirms results from Faulk and colleagues, who observed higher preservation of basement membrane in TritonX decellularized porcine urinary bladder compared to other DC reagents [43].

The ECM was biodegradable, which shows the possibility of scaffold remodeling by the patient’s own cells *in vivo*. Only the SDS group was significantly less affected by collagenase treatment. This could be explained by disruption of collagenase I cleavage sites by the SDS treatment and subsequently reduced enzyme activity, as observed in previous studies [28,44]. It is not expected that similar degradation kinetics will take place *in vivo*. However, the *in vitro* biodegradation assay can be seen as an accelerated model for graft remodeling [27,28,44–46].

The ECM integrity is closely related to the ECM stability. Biomechanical testing showed that the ECM stability (maximum tensile strength and burst pressure) was well preserved in all DC groups. Standard deviation both in the native and in the DC groups was high, even for ringlets originating from the same vein, indicating heterogeneous properties of the biological donor tissue. An increased stiffness was observed in the TX 1 group, possibly due to a long perfusion time (11 days). Other studies show reduced protein content when decellularizing bovine carotid arteries with different flow rates [14,47], indicating an effect of the perfusion application on mechanical properties. The stiffness of the ECM influences cell spreading, cell differentiation, and cell attachment to the biomaterial [48,49]. Increased ECM stiffness may negatively affect VEGF binding and uptake of endothelial cells [50]. Thus, DC vessels mimicking the native ECM in terms of elastic modulus is preferable for proper biocompatibility.

Acellular biomaterials often lead to thrombosis, intimal hyperplasia or aneurysms. Therefore recellularization or other preconditioning treatments prior to transplantation are seen as preferential [51]. Our data from the cell seeding experiment showed that the TX 1 and TX 2 protocols were better for recellularization with HUVECs, compared to SDS protocol, which is in line with previous reports [19,33,52]. While ionic detergents such as SDS and SDC fully denature proteins, non-ionic detergents such as TritonX keep native protein structures intact [15,19]. This might explain the improved recellularization potential in the TX groups. Furthermore, a previous study shows that the amount of vascular growth factors such as FGF and VEGF are not significantly decreased following TX decellularization of blood vessels [24], showing that TritonX treatment preserves growth factors. Hence, our results, as well as other studies, support that TX-based protocols are the most successful for recellularization of DC vessels with endothelial cells.

The biggest variations between the different protocols were observed in the DC and recellularization efficacy, and results from biodegradation, mechanical properties, ECM components and collagen degradation provided further valuable insights into DC optimization and can help to design a DC protocol for specific purposes. There is no consensus about what defines a successful decellularization, and previous publications have mentioned the importance of DNA removal observed by DNA quantification and histology [15]. This definition does not account for successful recellularization, which often is the final goal. Defining a standardized protocol for all decellularization processes seems difficult given the diverse intended applications and tissue types. Complete removal of cellular material and the ability of relevant cells to reattach to the DC tissue are important criteria. Overall, DC might help to overcome current limitations of synthetic materials and autologous grafts, yet defining the protein composition and interaction as well as surface structure which allows cells to attach to a biomaterial, differentiate and proliferate will be one of the main challenges for future studies [19]. This may allow the manufacturing of homogenous biomaterials for medical applications that don’t have the limitations of inhomogeneous biological tissues.

## Conclusion

In this study, we demonstrate the ability of 5 different protocols to fully decellularize porcine vena cava, and show that vessels decellularized with TritonX are most efficiently recellularized with HUVECs *in vitro*. Moreover, DNase application in combination with TritonX was demonstrated to be necessary for complete removal of cellular material.

## Supporting information

S1 Text Protocols for unsuccessful decellularization experiments of vena cava. (DOCX)

S1 Table Decellularization cost. Cost of decellularization of a single vein (€), only taken the detergent, chemical and enzyme costs into account. Usually, the cost of equipment and personnel exceeds the detergent costs, which is why total process time for one decellularization is a major economical factor, yet detergent cost has a greater role in upscale of process.
(DOCX)

S1 Fig Decellularized blood vessels stained with DAPI. Staining of sections of decellularized blood vessels with DAPI, showing remnant nucleic material in the groups TX 2 -DNase, TX 3 and TX 4. Scale Bar equals 100 μm.
(TIFF)

S2 Fig Histology of decellularized blood vessels. Histological stain of sections with Verhoeff van Giesson (VvG), Masson Trichrome (MTC), Hematoxylin-Eosin (H&E) Alcian Blue (AB) and Periodic Acid Schiff (PAS) staining. Scale Bar equals 100 μm for all.
(TIFF)

S3 Fig Biomechanical tests. Setup of the biomechanical tests. (A) In order to obtain equally sized vein ringlets, a device was constructed by connecting a commercial screw with multiple razor blades, separated with nuts and screw locking. (B) Veins were then pinned onto a wooden plate and cut into 3 rings per sample (C) by applying force with a hammer. (D) Elongation of the vein ringlets (dL) at maximum tensile strength (F_max_) shows the change in length in percentage compared to the starting length of the specimen. A trend towards increased dL was observed in the group TX 2 -DNase, while the groups CHAPS and TX 1–2 showed a significantly lower dL at the point of break.
(TIFF)

S4 Fig Stress strain curves of biomechanical tests. Stress strain curves of native and decellularized vessels are shown.
(TIFF)

## Abbreviations

AA: Antibiotic-Antimycotic
AB: Alcian Blue
CHAPS: 3-[(3-cholamidopropyl) dimethylammonio]-1-propane sulfonate
CHP: collagen hybridizing peptide
DAPI: 4',6-diamidino-2-phenylindole
DC: decellularized
Dnase: deoxyribonuclease
ECM: extracellular Matrix
GAG: glycosaminoglycan
H&E: Hematoxylin-Eosin
HUVEC: human umbilical vein endothelial cells
MTC: Masson Trichrome
n.s.: not significant
o/n: overnight
OD: optical density
SDC: sodium deoxycholate
SDS: sodium dodecyl sulfate
TNBP: tri-n-butyl-phosphate
VvG: Verhoeff van Gieson
