# Supplementary material for: Systematic in vitro comparison of decellularization protocols for blood vessels
Source: PLoS One. 2018 Dec 17;13(12):e0209269. doi: 10.1371/journal.pone.0209269 (PMC6296505; doi:10.1371/journal.pone.0209269)
Supplement: S1 Text — (DOCX) [file pone.0209269.s001.docx]

# **Supplementary Text 1:** **Protocols for unsuccessful decellularization experiments of vena cava**

TX 3: Incubation of vein in TNBP (1%) for 17h, followed by washing and incubation in Triton X (1%) for 3h. Washing and incubation continued in DNase (40 U/mL) for 2 h, followed by final wash (20h), sterilization (3h) and freezing.

TX 4: Incubation of vein in Triton X (1%) for 22h, followed by washing and incubation in TNBP (1%) for 22h. Washing of 1x 5 min in TritonX and 3x 5 min in H_2_O before incubated continued in DNase (40 U/mL) in PBS +/+ for 2 h, followed by final wash (7h), sterilization (70 min) and freezing.
