# Supplementary material for: Systematic in vitro comparison of decellularization protocols for blood vessels
Source: PLoS One. 2018 Dec 17;13(12):e0209269. doi: 10.1371/journal.pone.0209269 (PMC6296505; doi:10.1371/journal.pone.0209269)
Supplement: S1 Table — Cost of decellularization of a single vein (€), only taken the detergent, chemical and enzyme costs into account. Usually, the cost of equipment and personnel exceeds the detergent costs, which is why total process time for one decellularization is a major economical factor, yet detergent cost has a greater role in upscale of process. (DOCX) [file pone.0209269.s002.docx]

# **Supplementary Table 1: Decellularization Cost.**

| **Decellularization method** | **Cost/Vein (Euro, €)** |
| --- | --- |
| SDS | 16.59 |
| SDC | 20.09 |
| CHAPS | 21.41 |
| TX 1 | 98.13 |
| TX 2 | 32.67 |
| TX 2-DNase | 17.46 |

**Supplementary Table 1: Decellularization cost.** Cost of decellularization of a single vein (€), only taken the detergent, chemical and enzyme costs into account. Usually, the cost of equipment and personnel exceeds the detergent costs, which is why total process time for one decellularization is a major economical factor, yet detergent cost has a greater role in upscale of process.
