# Supplementary figures and images for: Systematic in vitro comparison of decellularization protocols for blood vessels
Source: PLoS One. 2018 Dec 17;13(12):e0209269. doi: 10.1371/journal.pone.0209269 (PMC6296505; doi:10.1371/journal.pone.0209269)

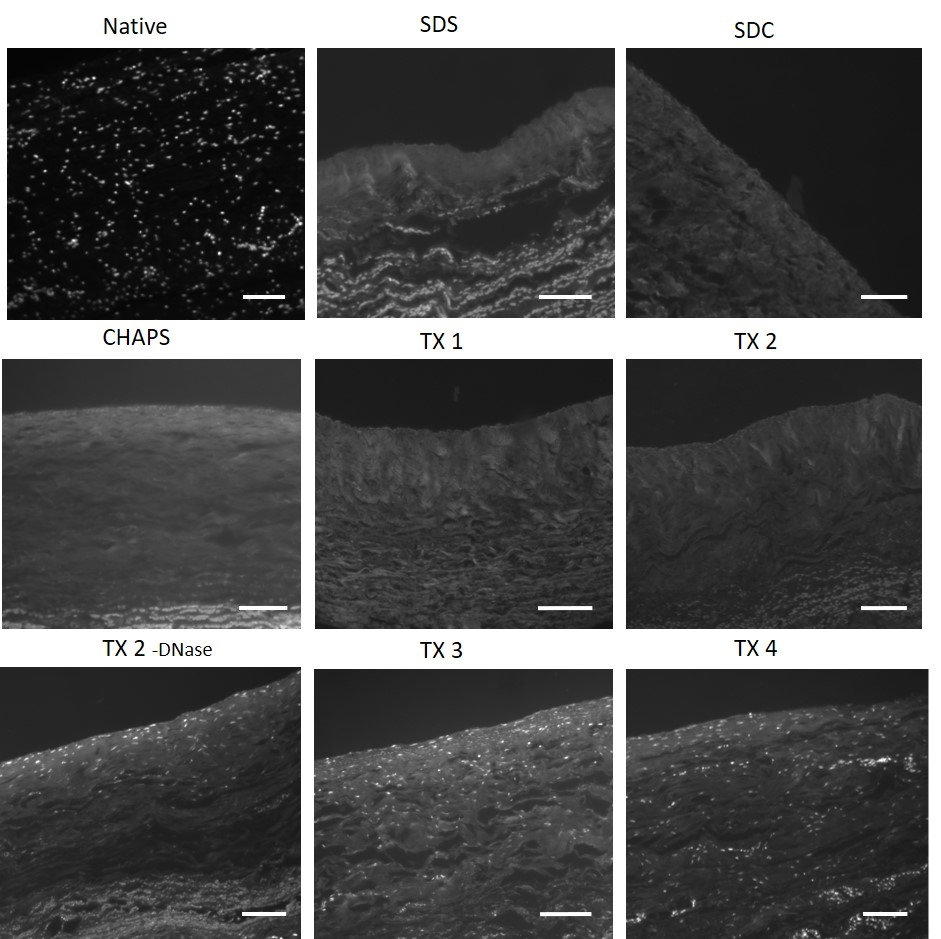

Supplement: S1 Fig — Staining of sections of decellularized blood vessels with DAPI, showing remnant nucleic material in the groups TX 2 -DNase, TX 3 and TX 4. Scale Bar equals 100 μm. (TIFF) [file pone.0209269.s003.tiff]

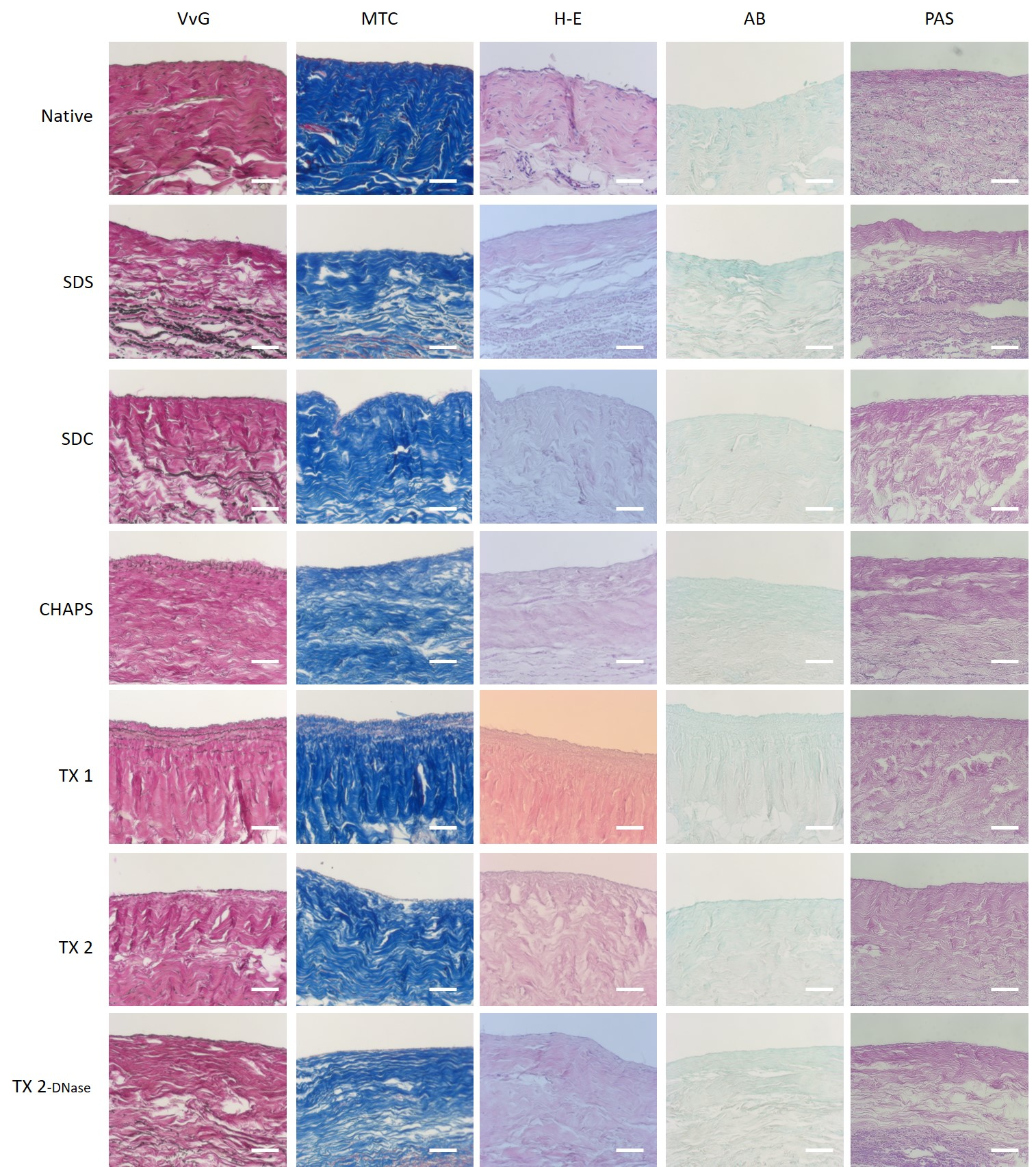

Supplement: S2 Fig — Histological stain of sections with Verhoeff van Giesson (VvG), Masson Trichrome (MTC), Hematoxylin-Eosin (H&E) Alcian Blue (AB) and Periodic Acid Schiff (PAS) staining. Scale Bar equals 100 μm for all. (TIFF) [file pone.0209269.s004.tiff]

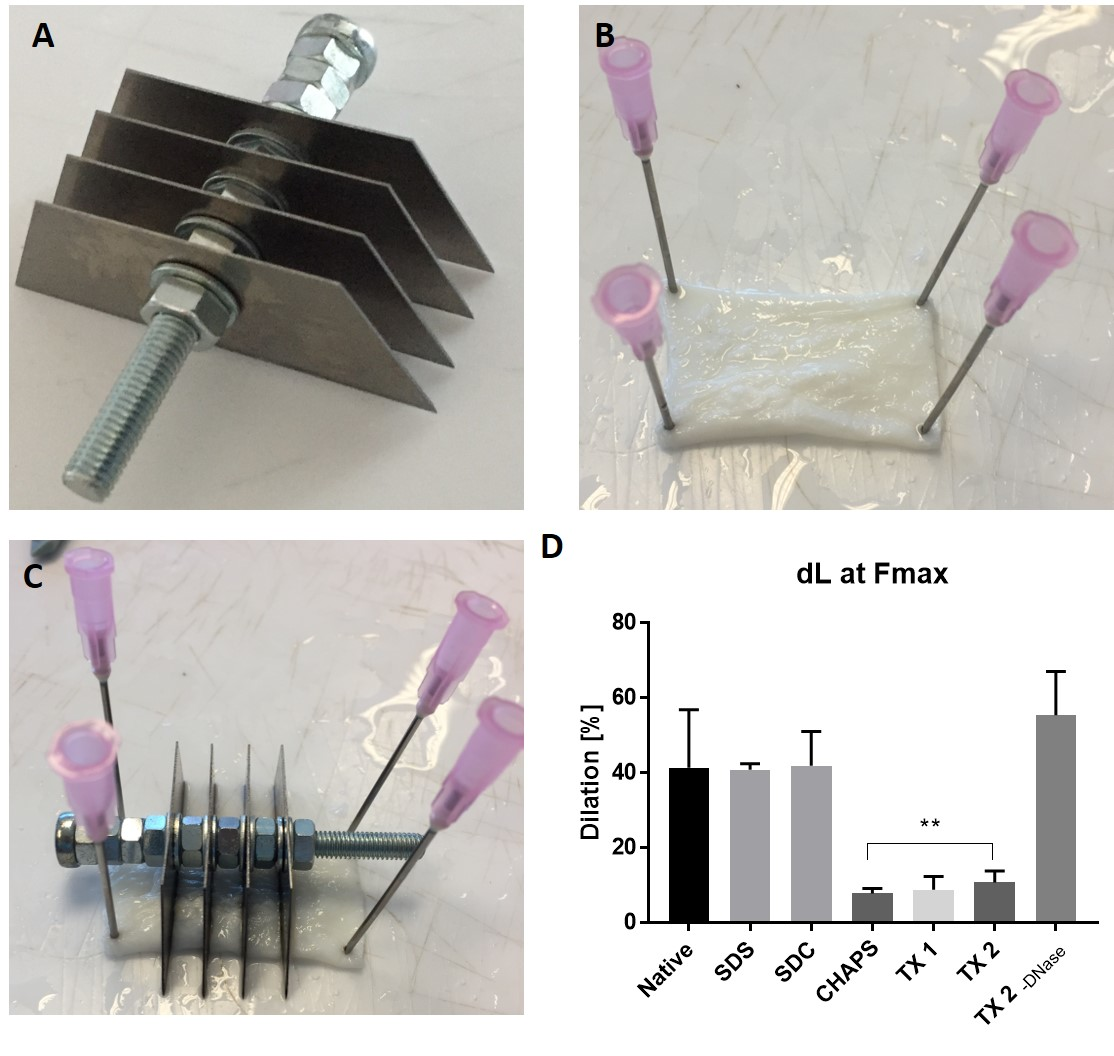

Supplement: S3 Fig — Setup of the biomechanical tests. (A) In order to obtain equally sized vein ringlets, a device was constructed by connecting a commercial screw with multiple razor blades, separated with nuts and screw locking. (B) Veins were then pinned onto a wooden plate and cut into 3 rings per sample (C) by applying force with a hammer. (D) Elongation of the vein ringlets (dL) at maximum tensile strength (Fmax) shows the change in length in percentage compared to the starting length of the specimen. A trend towards increased dL was observed in the group TX 2 -DNase, while the groups CHAPS and TX 1–2 showed a significantly lower dL at the point of break. (TIFF) [file pone.0209269.s005.tiff]

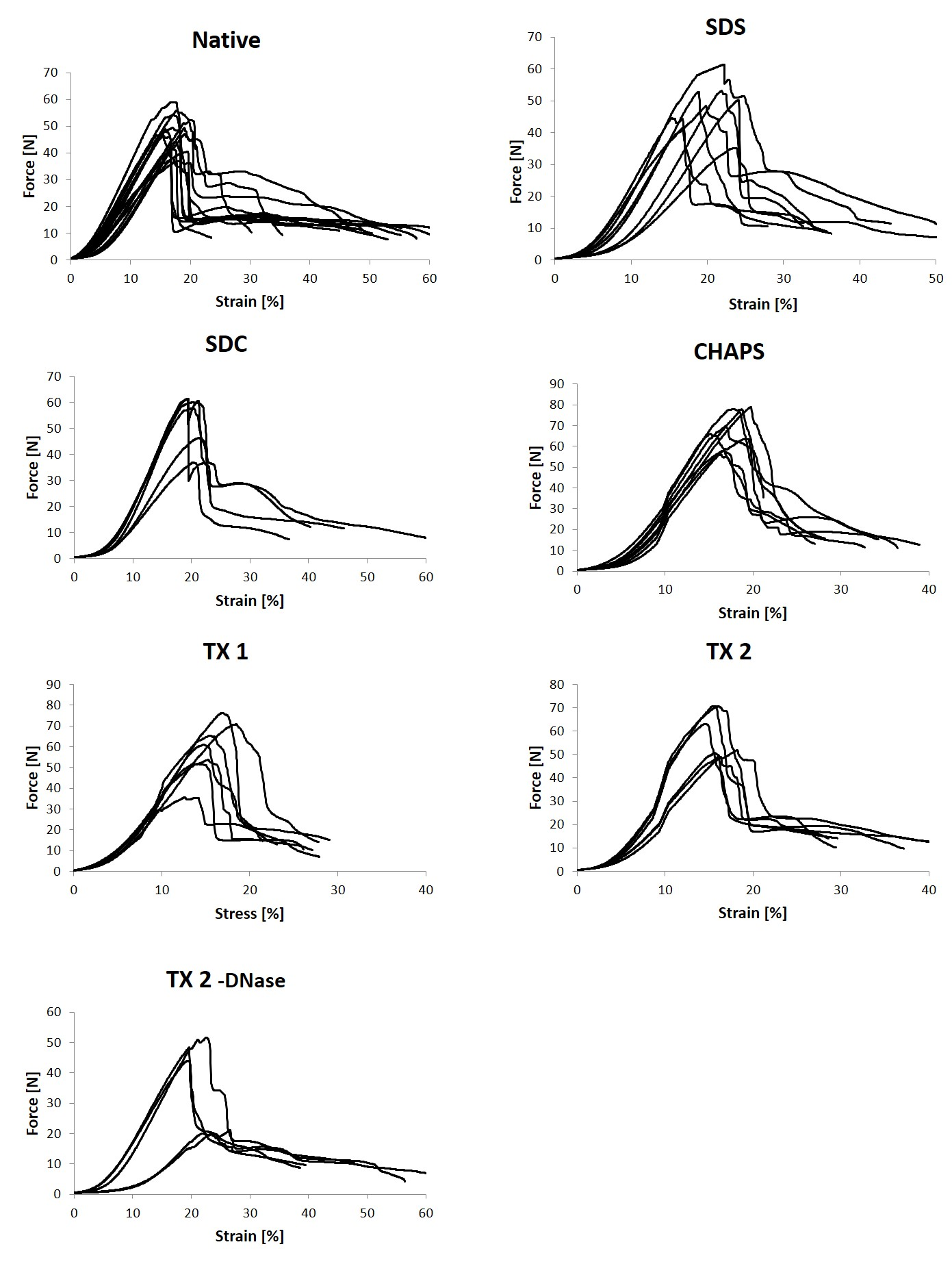

Supplement: S4 Fig — Stress strain curves of native and decellularized vessels are shown. (TIFF) [file pone.0209269.s006.tiff]
